# Multi-service prevention programs for pregnant and parenting women with substance use and multiple vulnerabilities: Program structure and clients’ perspectives on wraparound programming

**DOI:** 10.1186/s12884-020-03109-1

**Published:** 2020-08-03

**Authors:** Deborah Rutman, Carol Hubberstey, Nancy Poole, Rose A. Schmidt, Marilyn Van Bibber

**Affiliations:** 1 Principal, Nota Bene Consulting Group, 1434 Vining St, V8R 1P8 Victoria, BC Canada; 2 Centre of Excellence for Women’s Health, V6H 3N1 Vancouver, BC Canada

**Keywords:** gender, substance use, social determinants of health, FASD prevention, program evaluation, multi-service program delivery, client perspectives, service organization

## Abstract

**Background:**

In Canada, several community-based, multi-service programs aimed at reaching vulnerable pregnant or parenting women with substance use and complex issues have emerged. These programs offer basic needs and social supports along with perinatal, primary, and mental health care, as well as substance use services. Evaluations of these ‘one-stop’ programs have demonstrated positive outcomes; nevertheless, few published studies have focused on how these programs are structured, on their cross-sectoral partnerships, and on clients’ perceptions of their services.

**Methods:**

The *Co-Creating Evidence* (CCE) project was a three-year evaluation of eight multi-service programs located in six Canadian jurisdictions. The study used a mixed-methods design involving semi-structured interviews, questionnaires, output data, and de-identified client data. This article focuses on qualitative interviews undertaken with 125 clients during the first round of site visits, supplemented by interview data with program staff and service partners.

**Results:**

Each of the programs in the CCE study employs a multi-service model that both reflects a wrap-around approach to care and is intentionally geared to removing barriers to accessing services. The programs are either operated by a health authority (n = 4) or by a community-based agency (n = 4). The programs’ focus on the social determinants of health, and their provision of primary, prenatal, perinatal and mental health care services is essential; similarly, on-site substance use and trauma/violence related services is pivotal. Further, programs’ support in relation to women’s child welfare issues promotes collaboration, common understanding of expectations, and helps to prevent child/infant removals.

**Conclusions:**

The programs involved in the Co-Creating Evidence study have impressively blended social and primary care and prenatal care. Their success in respectfully and flexibly responding to women’s diverse needs, interests and readiness, within a community-based, wraparound service delivery model paves the way for others offering pre- and postnatal programming.

## Background

Women’s substance use is often intertwined with an array of issues, including physical, emotional, or sexual abuse, intimate partner violence, mental health concerns, child welfare involvement, physical health problems, and challenges related to social determinants of health such as low social support, deep poverty, precarious living conditions and homelessness [1–6]. Research contextualizing women’s substance use has identified pathways, including women’s experiences of trauma, partner violence, the child welfare system, racism, the impacts of colonization, mental health concerns and their mother’s use of alcohol during pregnancy [7]. 

Substance use during pregnancy is associated with risks of harm for both the fetus/infant and the mother; moreover, women’s substance use postnatally is associated with parenting difficulties and increased risk/rates of child removal [8]. At the same time, women with substance use concerns who are pregnant and/or parenting often encounter a number of barriers when seeking help, all the more so if they use substances and have multiple vulnerabilities. These have been well documented and include: feeling judged by others; fear of child welfare intervention; poor mental health supports; inadequate housing and transportation; lack of child care; and their partner’s substance use [1, 6, 9–11]. Systemic barriers also exist: substance use services and child protection services typically have operated discretely with their own set of goals, policies, philosophies, expectations, and legislative regulations and timelines, resulting in high rates of child apprehensions and reluctance to positively sanction parenting by women who use substances [12–14]. Nevertheless, pregnancy has been shown to be a pivotal time when women are interested in contemplating or making a significant life change, partly as a result of their desire to keep their newborn in their care and to regain custody of older children, and thus they are receptive to engaging with services [5, 15–19].

### Multi-service programs for women who are pregnant and/or parenting and use substances

Multi-service programs offering basic needs and social supports along with perinatal, primary, and mental health care, as well as substance use services are particularly effective for women who are otherwise wary of engaging with the formal systems of care [4, 5, 17, 20, 21]. Additionally, services that incorporate non-judgmental, relationship-based, trauma-informed and harm reduction approaches are considered best practice [4, 8, 16, 22, 23]. Women tend to respond more positively to programs that offer a multi-service, collaborative approach to services than they do to standard, single-service programs, citing non-judgmental attitudes, availability of reliable information, consistency of staff, reduced need to repeat their information, program accessibility, and high levels of material, emotional, and health related support as positive attributes of these programs [5, 15, 19, 24, 25].

In Canada, services designed to address the particular health needs of women who use substances and who are pregnant or parenting can operate in two different ways. Since the 1990s, several community-based, multi-service programs aimed at reaching vulnerable pregnant or parenting women with substance use and complex issues have emerged [4, 17, 18]. Offering a safe, single point of access, these programs rely on a range of formal and informal partnerships and community connections to reduce barriers and provide holistic care, including a variety of health, substance use and trauma-related services, children’s health services, nutrition and basic needs supports, drop-in and outreach, and in some cases, housing and child care [18]. Recent research has shown that women attending these programs were seeking support for multiple, interconnected concerns such as keeping their infant in their care, reducing/quitting substance use, and/or accessing safe housing [17].

As another approach, integrated treatment programs for women who are pregnant or parenting and their children have addictions treatment as the entry point and reduced substance use as the focal goal. These programs were designed to overcome the systemic barriers typically associated with more conventional fragmented service delivery structures and similarly offer a range of services that address women’s physical, mental, and social-economic well-being. [26, 27]

Evaluations of community-based women-centred, relationship-based, ‘one-stop’ approaches have demonstrated positive outcomes. Findings include increased prenatal visits, improved birth outcomes, reduced substance use, increased support and connection to services, improved health and wellness, improved housing, increased connection and/or custody of infants and children, and reduced isolation [4, 17, 19, 20, 28–32].

At the same time, few published studies have focused on how programs that serve pregnant and parenting women with substance use issues are structured, and limited information is available on the types of cross-sectoral partnerships necessary to support the work of these programs. Similarly, there is a paucity of research on clients’ perceptions of their care at these programs. A small literature exists in the context of integrated treatment programs: two studies in one Canadian province identified the most common networks of partnerships amongst integrated treatment programs and described the categories of services that exist as a result [25, 26]. In these studies, the most common cross-sectoral networks were between the integrated treatment programs and: substance use or mental health services; child protection services; parenting programming, developmental assessment and childcare; and other social services [26]. Less common partnerships included key forms of physician-based health care, such as primary care, prenatal care, and opiate agonist therapy, resulting in these services being only periodically available. [25, 26] There was considerable variability amongst the integrated treatment programs with respect to provision of services related to housing support, assistance with transportation, and therapeutic childcare, despite these services being perceived as important to achieving positive outcomes [25]. Nevertheless, both studies accentuated the importance of such connections in addressing the broad range of physical health and social determinants of health issues experienced by women with problematic substance use. Moreover, in Tarasoff and colleagues’ 2018 study [25], clients of integrated treatment programs perceived their care more positively than did clients of standard treatment programs, and a strong theme in clients’ comments was their appreciation of program staffs’ supportive and non-judgmental approach.

While these studies make a valuable contribution to the literature, the authors of this article know of no published studies that have focused specifically on the types of informal, formal or non-traditional partnerships that multi-service community-based agencies rely upon as means to deliver programming and services to highly marginalized women who are pregnant and/or parenting and using substances. Further, the authors know of no research highlighting clients’ perspectives on how they utilize the various services provided by holistic community-based services or how the care and supports they receive make a difference to them and their families.

In view of this knowledge gap, additional study of service delivery partnerships as well as clients’ use of and perspectives on the array of supports available through community-based multi-service programs is critical for program development and evaluation of ‘one-stop’ approaches to care for pregnant and parenting women with substance use and other complex concerns. This article aims to begin to address this knowledge gap through discussion of the services, partnerships and client perspectives of the eight Canadian programs involved in the Co-Creating Evidence project.

### Co-Creating Evidence Evaluation Project

**Fig. 1:**
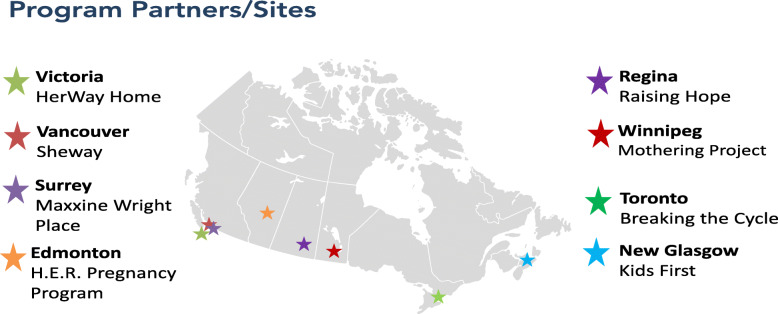
Co-Creating Evidence Project Program Sites/Locations

The *Co-Creating Evidence: National Evaluation of Multi-Service Programs Reaching Women at Risk* (CCE) project was a three-year evaluation of eight different holistic programs located in six Canadian jurisdictions, conducted between 2017 and 2020 (see Fig. 1). All eight programs serve highly vulnerable women at high risk of having an infant with prenatal substance exposure and/or affected by Fetal Alcohol Spectrum Disorder (FASD). At the same time, the programs participating in the project are not homogeneous. Each program’s services and supports have arisen out of its particular local context, including the cultural, geographical and socio-demographic environment and the community’s existing services, gaps, resources and partnership opportunities. While seven of the eight programs have the woman’s substance use as a primary eligibility criterion, they are all designed for women who are experiencing other concerns as well and thus include additional criteria. For example, one program is aimed at pre-natal and early parenting women impacted by both substance and/or violence; another one addresses homelessness along with substance use; while a third program is aimed at women under age 24 who need additional support with child protection, isolation, substance use, the effects of poverty, and navigating systems. That said, all programs employ relationship-based, trauma-informed and harm reduction approaches in line with good practice.

The goals of the project were to: share practice knowledge; demonstrate the effectiveness of prevention programming serving women with substance use and complex issues; and identify characteristics that make these programs successful. The eight program sites volunteered to be part of the study.

All eight programs are specifically for women, and for all programs, the focal client is the woman or the woman and her child. That said, at one program, women’s partners can attend the daily drop-in lunches, and at another program, partners can attend a weekly drop-in group with participants’ consent; two other programs are located within a community-based agency that is accessible to all genders. Seven of the eight programs serve pregnant or early parenting women with substance use issues and/or other complex challenges; the eighth program, located in a region with very few services, focuses on pregnant/parenting women who are at risk by virtue of being young (age 16–24) and possibly socially isolated.

Each of the eight programs participating in the CCE study employs a multi-service model that both reflects a wrap-around approach to care and is intentionally geared to removing barriers to accessing services and to providing services identified in the literature as meeting women’s holistic needs. Table 1  depicts the array of services provided on-site by the eight programs taking part in the CCE study. These services were delivered via: program staff; a combination of in-kind contributions, contract, or co-located services; or in the community by way of formal or informal partnerships. Taken together, the services comprised key elements of the wrap-around approach and are part of programs’ regularly scheduled activities.

**Table 1:**
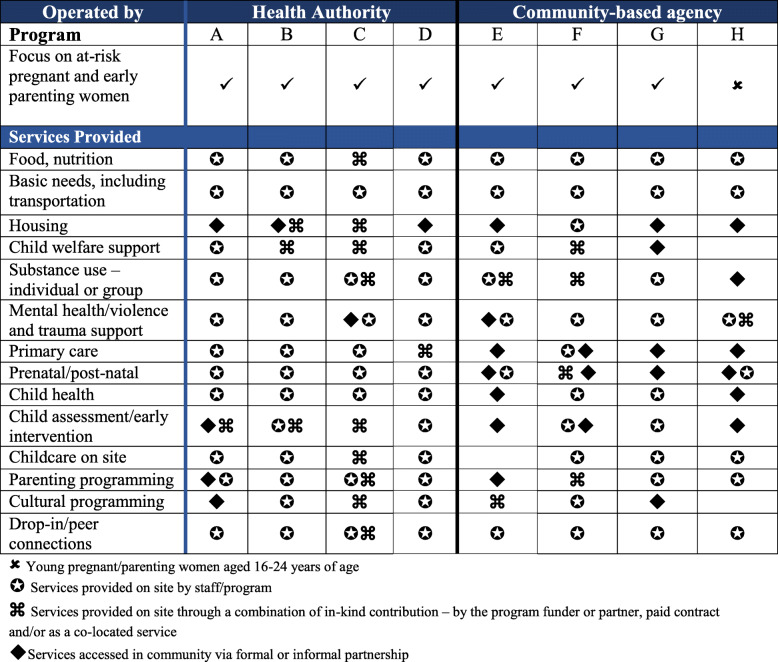
Services and activities offered on-site by program staff, co-located services, or in-kind or contracted services

### Study’s objectives and research questions

The purpose of this article is to describe the array of wraparound services and supports offered by the eight programs participating in the Co-Creating Evidence study, and how the programs organize their services to facilitate access to ‘one-stop’ health and social care. In addition, this article explores how clients utilize these health, housing, food-related, child welfare, parenting, childcare, cultural, trauma-related and substance use services and supports, highlighting clients’ own descriptions of the programming, how they engage with services, and how the supports/services make a difference to them and their children.

The article addresses the following research questions:

- How are the CCE programs’ wraparound services delivered – i.e., through core staff, contracted services and/or program partnerships?
- How do clients describe their utilization of and involvement with services, and how do the services and programming make a difference?

## Methods

### Study Design

The Co-Creating Evidence study is guided by principles of collaboration [32], including the principles of fostering meaningful partnerships and relationships with program staff and stakeholders, promoting participatory processes, developing a shared understanding of the programs, and fostering evaluative thinking [33, 34]. In keeping with these principles, as an initial project activity, in June 2017 the project team convened an introductory day-long in-person meeting with the eight program leaders in order to collaboratively identify a theory of change and to articulate the theoretical/philosophical foundations, approaches, key activities, and anticipated outcomes of the programs collectively. Bi-monthly web-based teleconferences have been held with program sites since then to discuss key issues related to data collection and analysis and to solicit the programs’ feedback regarding emerging project findings and knowledge translation; a second in-person meeting with all the programs took place to wrap up the project in 2020. A national Advisory Committee was created at the beginning of the project and has met 2–3 times a year to provide guidance and feedback on key facets of the project, including data collection and knowledge translation. For more information about the study design, see [5].

The Co-Creating Evidence study used a mixed-methods design involving semi-structured interviews, questionnaires, focus groups, output/program data, and client intake/outcome ‘snapshot’ data. Data were gathered in two ways: 1) by the project team, who conducted face-to-face, semi-structured interviews, focus groups, and questionnaires with clients, program staff and service partners in spring 2018 and fall 2019; and 2) by the program sites, who collected quantitative output data and client-based data from April 2018 through September 2019.

The study received ethics approval from the University of British Columbia Office of Research Ethics (H17-02168), Vancouver Costal Health Authority, Island Health Authority, Fraser Health Authority, and York University. All participants gave written informed consent to take part in the study; all were over age 18 and were competent to give their own consent.

### Data Collection Processes and Instruments

This article focuses on data from qualitative interviews undertaken with clients during the first round of site visits between April and July 2018. It is supplemented by interview and focus group data with program staff and the programs’ service partners.

Interviews and focus groups with program staff focused on staffs’ and managers’ perspectives on program goals, foundational principles and approaches, program operational issues (e.g., staffing, training, supervision, funding), program partnerships, and program impacts for clients, families and community partners. Interviews with program partners focused on partners’ perspectives on the partnership and any practice-related and organizational impacts of the partnership, as well as partners’ perspectives on the program’s strengths, challenges, and outcomes. Interviews with clients were conducted using a guided conversation approach that enabled interview participants to speak freely about whatever was most important to them. The client interview guide contained open-ended questions focusing on: the woman’s life situation at the point at which she first engaged with the program and what she hoped to get from her involvement with it; whether and how she utilized the different services and types of programming offered by her program; her satisfaction with the program; and what had been the most significant change for her and/or her family since her involvement with her program. Immediately following the interview, women were invited to complete the Client Questionnaire, which was most often administered verbally by the research team member. Additional information about on-site data collection by the project team is provided elsewhere (see [5, 17]).

### Participants

A total of 125 clients participated in the first round of in-person data collection for the study. In addition, a total of 61 program staff took part in interviews or focus groups at the eight programs; 42 service partners were also interviewed. Based on questionnaire data, all clients who participated in face-to-face interviews and completed the Client Questionnaire identified their gender as female, and more than half (54%) were older than 30 years old. The majority of clients (53%) self-identified their cultural background as Indigenous, followed by European/White (27%) and mixed race (15%), though this varied across programs from 97% at the Indigenous-focused program in downtown Winnipeg to 0% at the program in rural Nova Scotia. The length of time women had participated in their program varied from less than one month to more than three years. This variation in part is attributable to the policies and funding realities of the individual programs; for example, clients can participate in several programs till their child is school-aged, whereas at other programs, clients end their formal involvement at 6-months or 12-months post-partum.

Across the eight CCE programs, the 61 program staff who participated in interviews or a focus group came from diverse professional backgrounds, and included: the manager or coordinator of each program, nurses, physicians, nurse practitioners, midwives, social workers, outreach workers, counsellors, client engagement workers, Elders, Indigenous liaison workers, infant development workers, peer support workers, Aboriginal family support workers, childcare workers, and the executive directors of programs’ sponsoring organizations.

### Data Analysis

For the interviews with clients, program staff and services partners, qualitative data analysis techniques were utilized; NVivo12 software (QRS International, Melbourne, Australia), was utilized to facilitate the analyses. See [5, 17] for additional information about the study’s data analysis techniques.

## Results

### Wrap-around services offered by the CCE programs: How services are delivered through the program’s staffing and via partnerships

The programs participating in the Co-Creating Evidence study are operated (and largely funded) by a health authority (*n* = 4) or by a community-based agency (i.e., non-profit organization with funding from a range of local, provincial, and federal funding sources) (*n* = 4). Programs operating through a health authority were more likely to include ready access to a wide range of health services on-site, such as: public health nursing; nurse practitioner; physician; specialist addictions and maternity care, obstetrics, and maternal-fetal medicine; psychiatry; or midwifery. Overall this meant that women typically received both regular and specialized medical care that they would otherwise have a difficult time accessing in the community. As described by program staff:

*We also have two addictions doctors who come twice a week. The doctor is really important for women, because they wouldn’t be able to access that in the same way in the community, especially having access to the addictions doctor. There’s also medical coverage during the prenatal and postnatal groups.*

That said, one of the programs operated by a non-profit agency offered ready access to *primary health care* and individual and group-based substance use and mental health services through contracted services with a physician and a psychologist, as well as partnerships with the health authority’s mental health and addictions services. Being situated on-site helped raise practitioners’ visibility, while allowing more time to gain trust and reduce women’s anxiety about accessing health care.

The other three programs operating through a community agency also offered regular access to some aspects of primary health care, although this was delivered primarily in the community and not on-site at the program. For example, one program provided limited health care in the form of on-site pregnancy testing and monitoring and testing for sexually transmitted infections. This program also worked collaboratively with two community-based interdisciplinary primary health care centres serving people with complex needs. This partnership arrangement allowed for sharing information about mutual clients. According to the staff at this program, most of the clients were wary of formalized health care, thus the collaborative relationship with the health centres made it more likely that clients would access and receive health care. As one health care partner characterized the relationship:

*They (the program staff) coordinate services for their women and are there to be a bigger participant in the women’s pre- and postnatal care. The biggest strength is to have a close working relationship with the shared goal to support marginalized women with their pregnancy.*

Through Memorandum of Understandings, another program in the study forged working relationships with a handful of specialized programs for high risk and/or substance using pregnant women, thereby giving its clients access to specialized prenatal care and again making visible women’s unique health care requirements when substance use was also an issue.

Seven of the eight programs taking part in the Co-Creating Evidence study actively work with women to address child safety concerns. Having an alliance or partnership with *child welfare services* was another important achievement; as noted in Table 1, several programs had a social worker with knowledge of provincial child welfare regulations on site – either as part of core staff or through an agreement with provincial child welfare services. At three programs, staffing includes an on-site social worker or case manager who provides clients with child welfare related information, support and advocacy; an additional four programs have service partnerships wherein a child welfare worker from the provincial child and family services ministry liaises with or comes into the program regularly to provide information and help to clients so that they can navigate extant child safety expectations and concerns.

This connection was valuable as it had the potential to positively impact how child welfare services and other service providers viewed a women’s capacity to parent. When government social workers were co-located at the program they could function as a bridge between the program and child protection services, a practice that in the words of social workers could result in everyone having a clearer idea of what was required for the women to achieve their goal of keeping their child:

*These women have experienced trauma, and the program works with them on planning for reunification. However, given child protection concerns, the plans have to be done in a careful way. Having our social worker at the program helps bridge the gap between us and the women.*

In addition, government child protection social workers who were involved at a case conferencing or planning level reported that through their relationship with program staff, they became more knowledgeable about the factors influencing women’s lives and therefore could have a more nuanced discussion about options and strategies for keeping children safe:

*People really listen and seek out the [program’s] staff. We go to them and ask, ”Help me understand what the woman’s experience is.” Staff bring back a broader perspective and understanding around trauma. Our workers are less blaming; they are recognizing that what we are seeing is a result of trauma rather than intentional non-compliance.*

Program managers also sought connections with complementary services and programming in areas other than health care, substance use, and child welfare services. Several programs were able to link their clients to services in the community by means of collaborative working relationships or formal partnerships. Two programs formed close working relationships with local Indigenous agencies so that their clients would have access to relevant *cultural services and programming*. The same was true of *housing*, which was a major challenge at all program sites. In addition to the three programs that had a direct link to supportive housing, one program had an informal partnership with a supported housing program geared to pregnant women at high risk of experiencing homelessness, and another program offered rental supplements to some clients. Program managers also sought ways to strengthen their on-site services by building or deepening relationships with service providers who had expertise in areas that could benefit the program and clients. As one manager explained:

*We also would like to have more involvement from someone with expertise in children’s behaviours, so that we’d be able to do some educational programming for women about children’s behaviours through the reunification process. We have a meeting with [a community program] about this possibility.*

### Wrap-around services offered by the CCE programs: What is offered and how do clients engage with their program’s services and supports

**Fig. 2:**
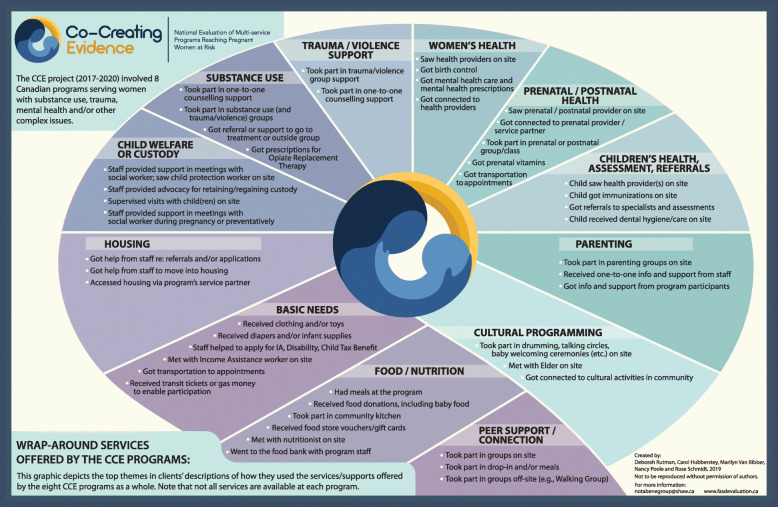
Clients’ descriptions of how they utilized the services offered by their program

In the interviews, clients were asked how they utilized their program’s array of services and supports. Figure 2 depicts clients’ descriptions of the ways in which they engaged with their program’s services. While it is important to note that not all services or types of programming are available at each program and that there is considerable variability in the ways and degree to which clients access the different services and supports offered at their program - as is in keeping with a client-centred approach to service delivery - what is striking about Fig. 2 is the holistic array of services and supports available at these programs overall.

**Fig. 3:**
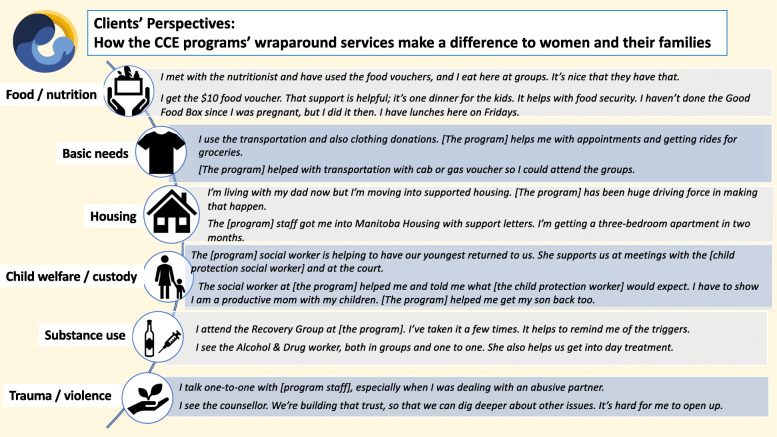
How clients engaged with programming and how services made a difference (Part A). Legend: Source for Food security icon: The Noun Project made available under Creative Commons. All other icons shown in this figure were accessed through PowerPoint software

**Fig. 4:**
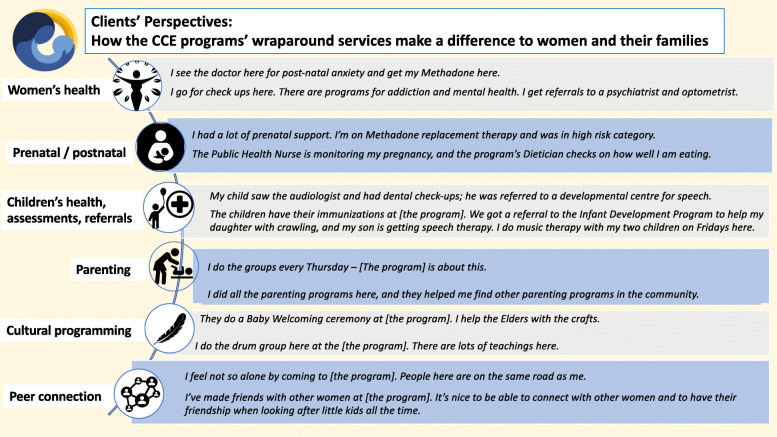
How clients engaged with programming and how services made a difference (Part B). Legend: Source for Women’s health icon: Icon made by Freepak from flatiron.com. Source for Prenatal/postnatal icon: “Breastfeeding” by Edward Boatman, US. Public domain. Source for Cultural programming feather icon: B Barrett, The Noun Project, made available under Creative commons. All other icons shown in this figure were accessed through PowerPoint software

By describing their use of their program’s services and programming in their own words, clients could also elaborate on the ways in which they were involved with the services and supports, as well as how the services made a difference to them. A sample of women’s comments is presented in the Figs. 3 and 4.

Qualitative analyses of clients’ comments regarding how they engaged with services and why they were helpful resulted in several key themes, including:

- Programs’ focus on the social determinants of health is paramount
- Wrap-around services help ensure women access a wide range of needed primary care, as well as prenatal, postnatal and mental health care
- On-site substance use and trauma/violence counselling and support make a pivotal difference
- Programs’ support in relation to women’s child welfare issues promotes collaboration, common understanding of expectations, and helps to prevent child/infant removals
- Programs’ developmental lens helps women access key parenting and pediatric services – i.e., services for themselves, for their child(ren), and for the mother-child connection
- Cultural programming promotes women’s (re)connection to traditional knowledge and teachings, and to holistic and land-based healing practices

Brief discussion of these themes follows.

#### Programs’ focus on the social determinants of health is paramount

The programs’ use of a social determinants of health lens in understanding women’s needs is a key factor in their ability to engage with women. Food/nutrition-related programming and basic needs support contribute in fundamental ways to the women’s health and to positive birth outcomes, mitigate women’s poverty, and bolster women’s capacity to keep their infant/child(ren) in their care. Many clients also spoke of being drawn to their program because of the meals and other basic needs supports, yet then engaging with program staff and staying on, accessing the program’s array of services. In women’s words:

*They helped me with basics needs. I couldn’t afford groceries, so I used the Community Cupboard – I used it for groceries for two weeks a month for three months.*

*The food, the doctors. Health care is the best in the city. The services and supports – the staff are kind and non-judgmental.*

In their comments about the food- and nutrition-related programming, a number of women also expressed their appreciation for their program’s provision of healthy food, which they valued for themselves and their children especially given that nutritious food can be too costly on a limited budget. As well, food-related programming offered opportunities for social connection, peer support and mentoring, and skill development, all of which were linked to wellness and personal development outcomes for women.

*They gave me food vouchers and the hot meals at drop in groups. We talked about different ways to introduce different kinds of food to children.*

*I get the Food Bank deliveries. I go to the cooking class here. We do potlucks. We all cook and share something. Our big thing here is that it takes a community. We all try to help each other out.*

Similarly, the programs’ focus on addressing women’s housing needs and assisting them to access safe and stable housing is especially important, given the inter-connections between housing adequacy and child welfare/protection involvement. Many clients reported that having safe and adequate housing was a key precondition for resolving concerns about child(ren)’s safety. Conversely, for some clients, not having safe housing was a factor in their infant’s or child’s removal from their care.

*They helped me in getting into ‘Moms and babies’ housing. It’s important because it is hard to get into housing that is Ministry-approved, so moms can keep baby with them.*

*[Program] staff are helping me to apply for housing. Without housing, I can’t get my baby back.*

### Wrap-around services help ensure women access a wide range of needed primary care, as well as prenatal, postnatal and mental health care

A strong theme in clients’ comments was that having an array of health and social care services under one roof helped to ensure that they got to their various appointments, participated in programming, and were able to address multiple needs at the same time. For example, this client stated:

*My doctor is at [the program]. The appointments are easier to keep; I’m not being referred to health services in [another community], which is what was happening when I lived in Vancouver. I couldn’t get to the appointments reliably. Now I’m able to, and I’m getting bloodwork done.*

As a related point, clients expressed appreciation for the trusting relationships they developed with care providers who knew of and were sensitive to their personal histories:

*I’m still connected with the Nurse Practitioner here. She’s the only heath care provider I’ll see because she knows me and my history.*

Combining on-site services with transportation and accompaniment by program staff also helped to ensure that clients successfully accessed important health and prenatal/postnatal appointments:

*I go to the Thursday night classes, and they connected me to my midwife and take me to my appointments.*

#### On-site substance use and trauma/violence counselling and support make a pivotal difference

Programs with on-site group-based and/or one-to-one substance use counselling and support, coupled with on-site trauma/violence-related programming made a big difference for women. It is important to reiterate that these are not integrated treatment programs, and women do not come to these programs solely for substance use or trauma-related services. That said, the majority of clients with substance use concerns come to their program in a place of readiness to take steps to reduce or quit their problematic substance use, and they value the groups and individual support they receive:

*I do one-to-one counselling with [program staff person] who helped me to get clear about relationships and alcohol. I also attended the program’s Recovery Group.*

*I’ve talked with the midwife here. I wouldn’t be still clean and sober if it weren’t for the support here. Sometimes I’m doing well, sometimes not. But I don’t do alcohol and drugs when I’m having a bad day. It’s about maintaining your life.*

Moreover, clients expressed that the approaches used by program staff (e.g., relational, harm reduction, trauma-informed, culturally safe, etc.) promoted a sense of safety, honesty, trust and community. These approaches were especially important given that so many of the women served by these programs had previously had negative experiences with health and social care systems and hence often avoided them. As these two clients stated:

*I quit cold turkey from alcohol and drugs when I was five and a half months pregnant with my daughter. I talk to [staff person] about everything, even about my urges to use.*

*I have seen the counsellor a few times. I quit while pregnant. I haven’t been ready to go to a group. It’s the first time I’ve been sober for this long since I was 12 years old. Just having a safe space to go to where no one is drinking really helps, to be in a community that sees me as a mom and not as an alcoholic.*

### Programs’ support in relation to women’s child welfare issues promotes collaboration, common understanding of expectations, and helps to prevent child/infant removals

In describing their use of their programs’ child welfare related services/supports, many clients expressed deep appreciation for the assistance, accompaniment and advocacy they received from program staff. Many also stated that they believed that the program’s support made a critical difference in enabling them to keep their infant and/or regain their older children to their care. In these clients’ words:

*[The program’s] social worker made the appointment for me to meet with the Child and Family Services worker. I had to stay clean and sober and get a restraining order against my partner. Because of that, Child and Family Services did not flag me, and I had no issues in the hospital.*

*[The program’s] social worker helped me to meet with [the government’s] child protection workers. She explained to me where they were coming from and she could tell the [government’s] social workers what I was feeling. [The program] staff were always with me, so I was supported and able to go to court for custody.*

### Programs’ developmental lens helps women access key pediatric and parenting services – i.e., for themselves, for their infant/children, and for the mother-child connection

Seven of the eight programs involved in the study offered key pediatric services (e.g., immunizations, well-baby check-ups and/or routine developmental assessments) and parenting- related programming on-site either by program staff or by a co-located service partner. Moreover, one of the programs, which defined itself as children’s developmental assessment and early intervention program with wrap-around services for women, had a dual-focus model wherein both mother and her infant/child were considered program clients; an eligibility criterion for this program was that women had to have the goal of actively parenting their infant. At the same time, clients participating in nearly all of the Co-Creating Evidence programs voiced their appreciation of their program’s health and developmental services for their infant/child(ren).

*The children have their immunizations at [the program]. We got a referral to the Infant Development Program to help my daughter with crawling, and my son is getting speech therapy. I do music therapy with my two children on Fridays at [the program].*

*I do groups and have at home visits with the parenting intervention therapist from [the program]. These were the best because they could see me at home and at ease with my children.*

### Cultural programming promotes women’s (re)connection to traditional knowledge and teachings, and to holistic and land-based healing practices

Five of the eight programs involved in the Co-creating Evidence study engaged an Indigenous Elder or Cultural Liaison who came to the program or was available to clients at least once a week for group-based programming (e.g., drumming, Talking Circles, traditional parenting), ceremonies, smudging, one-to-one meetings and/or who connected women with cultural activities in the community (e.g., Round Dances, sweats) or on the land (e.g., gathering medicines, sage, sweetgrass, berries). Clients expressed their appreciation of the programs offering various opportunities to women to be introduced to or re-engage with culture and traditional ceremonies; similarly, programs having both group-based programming, including activities that could involve the women’s children, and one-to-one time was valued.

*We do drumming. We’ve made drums. My son loves drumming. We had a baby-welcoming ceremony and smudges.*

*The Elders that they have brought in here are great. I like the baby welcoming ceremony.*

## Discussion

The current study illustrates that women’s and children’s lives span boundaries and cannot be easily compartmentalized into traditional funding and service delivery structures. Consequently, a multi-sector, multi-service, wrap around programming and partnership approach is essential.

When it comes to key activities and services, all programs employ some combination of primary, prenatal, perinatal and mental health care services; moreover, the programs are flexible and evolving, adding and adapting services and supports as women’s and their families’ needs are identified and programming / partnership opportunities emerge, yet adjusting levels of service when funding does not keep pace with costs. Being connected with a range of health care providers paves the way for women to receive multi-dimensional health care and for their children to receive developmental screening and follow-up. How this is operationalized is not as important as the fact that women made a positive attachment with a health care provider, whether that is a nurse, nurse practitioner, physician, maternal care or addictions specialist, midwife, psychiatrist, psychologist or counselor. Women’s engagement with health services in turn results in their children receiving important assessment and pediatric attention. When health services have been offered in-house the connections are more readily facilitated, but it can be seen that programs that relied on off-site providers are also able to effectively help clients to access and engage with services by virtue of the strong relationships between the program and its partners.

The ability of the programs to support and strengthen the mother-child connection is an important outcome. Inclusion of child welfare support is thus another key component of the wraparound programs, as it helps women keep their infant and/or regain their children and helps government child protection workers gain a deeper appreciation of women’s capacity to parent, their support needs, and an understanding of how relational, harm reduction and trauma-informed approaches work in practice [20].

An approach designed to meet the wide range of women’s material and social needs and to address barriers to service is similarly fundamental to the programs’ and women’s success. Offering a variety of programming related to food and nutrition was a much-valued means to facilitate and sustain women’s engagement in their program, frequently leading to important peer connections and supports, and becoming an entry point to health and prenatal care. The provision of childcare similarly facilitated clients’ participation in key group-based programming and/or enabled women to access services or attend appointments in the community. For several of the programs in the Co-Creating Evidence study, funding for these types of activities came from community sources such as a local charitable foundation, food bank, non-profit community group, or educational institution. This has the advantage of helping to promote a connection between the programs and their communities that is also reflective of their local context. It also affords program managers leeway in not having to draw down on already stretched core funding while still meeting needs associated with determinants of health.

In addition to the wide-ranging array of services/activities, the relational approaches taken by the program staff are also key. Trauma-informed, non-judgmental and non-stigmatizing approaches that recognize the importance of women’s safety, choice, and readiness to set goals and make changes in their lives exemplify ways of successfully engaging women in programs. These approaches are also consistent with the literature, which has demonstrated that a relational and non-judgmental approaches are foundational to constructive change, be that at the individual, institutional or systemic level [18, 22, 35].

Moreover, in keeping with a women-centred approach, women choose what services/programming they access. Services and programming are tailored to known goals, needs and circumstances of pregnant and parenting women who use substances; however, within that evidence-informed framework, women have agency to access what services they feel will work for them, at the pace for which they are ready. This aspect of the services, as illustrated in Fig. 1, is highly important to the structuring of these programs, indicative of a power-with rather than a power-over approach. For women with histories and current experiences of trauma and violence, significant exposure to stigmatizing attitudes for their use of substances, and overall powerlessness in many interactions with systems of care, this aspect of their structure is reparative, welcoming and respectful. To some women this is a first encounter with such respectful and empowering service interactions.

### Study Limitations

This multi-site evaluation study involved eight programs that all volunteered to participate, and the on-site client-related data collection (i.e., interviews with clients) also employed a volunteer sampling approach. We recognize that that approach could have resulted in bias, in that clients with more involvement in and/or positive views about their program could have been disproportionately inclined to take part in the evaluation study. Nevertheless, as this article focuses on describing the programs’ structure and clients’ perspectives on wraparound programming, we have no reason to believe that clients having less positive perspectives were disinclined to participate in the study. Moreover, the guided conversational approach to interviewing facilitated participants to share their diverse experiences and perspectives.

## Conclusion

The programs involved in the Co-Creating Evidence study have assiduously blended social, primary, and prenatal care. Although not all programs provide all of the identified services or focus on providing all of the services to the same degree, through a combination of co-location with other services, shared services and staff, and relationships with service partners, they all offer a mix of services and supports. These services are provided either on-site or by connecting women to a wide array of services and support. Indeed, their ability to blend social and health care services is a distinguishing feature of these types of programs. They have cut through barriers that traditionally have seemed insurmountable, to provide context-specific support to pregnant women and new mothers and children that address a very wide range of social and structural determinants of health. Their success in respectfully and flexibly responding to women’s diverse needs, interests and readiness, within a community-based, wraparound service delivery model paves the way for others offering pre and postnatal programming to follow.

## Data Availability

The data produced and analysed during the current study are not publicly available, in keeping with the study’s approved ethical protocols.

## Abbreviations

CCE: Co-Creating Evidence project
FASD: Fetal Alcohol Spectrum Disorder
